# Tumor Budding in Gynecologic Cancer as a Marker for Poor Survival: A Systematic Review and Meta-Analysis of the Perspectives of Epithelial–Mesenchymal Transition

**DOI:** 10.3390/cancers14061431

**Published:** 2022-03-10

**Authors:** Muhammad Joan Ailia, Nishant Thakur, Yosep Chong, Kwangil Yim

**Affiliations:** Department of Hospital Pathology, College of Medicine, The Catholic University of Korea, Seoul 06591, Korea; vetjoan@gmail.com (M.J.A.); nishantbiotech2014@gmail.com (N.T.); ychong@catholic.ac.kr (Y.C.)

**Keywords:** gynecologic cancer, tumor budding, prognosis, pathology, systematic review

## Abstract

**Simple Summary:**

Tumor budding (TB) is an emerging prognostic marker in various cancers; specifically, its role is well established in colorectal cancer. There are very few studies on TB’s role in gynecological cancers. Thus, we studied tumor budding relationships with gynecological cancers and tried to figure out its role in patient survival outcomes. Total eleven cohort studies (seven cervical and four endometrial cancers) were enrolled. TB showed a poor prognosis in terms of survival and clinicopathological parameters outcome. TB was related to epithelial–mesenchymal transition, microvessel density, and decreased hormone receptor expression. TB can be used as future prognostic marker in gynecologic cancers.

**Abstract:**

This study aimed to assess the prognostic significance, assessment methods, and molecular features of tumor budding (TB). A literature search of Medline, EMBASE, Cochrane Library, and eleven cohort studies (seven cervical and four endometrial cancers) was conducted. Three assessment methods for TB involving 2009 patients were collected and constituted in the analysis. Our meta-analysis showed that TB was a marker of poor survival, regardless of the cancer origin site or assessment method (overall survival: hazard ratio [HR], 2.40; 95% confidence interval [CI], 1.82–3.17; disease-free survival: HR, 3.32; 95% CI, 2.46–4.48). In endometrial cancers, TB is associated with the epithelial–mesenchymal transition, microvessel density, and decreased hormone receptor expression. Thus, we suggest TB as a poor prognostic marker for all gynecologic cancers.

## 1. Introduction

Gynecologic malignancies are the leading cause of cancer-related death worldwide [1,2]. Several effective treatment methods exist for gynecological cancers, such as adjuvant chemotherapy and targeted therapy [3]. However, owing to their heterogeneity, selecting the appropriate treatment for individual patients is important. Therefore, clinical or pathological prognostic predictors are required.

Recently, tumor budding (TB) has evolved as a novel marker for poor prognosis and epithelial–mesenchymal transition (EMT) in several solid tumors [4,5]. TB is defined as isolated single cancer cells or clusters of up to four cancer cells at the invasive tumor front [4]. TB is a poor prognostic factor of survival that is correlated with clinical predictive factors such as T stage, lymph node metastasis (LNM), and lymphovascular invasion (LVI) in colorectal adenocarcinomas [6,7,8]. Emerging data suggest that TB is also correlated with such factors in gastric cancer (T stage, LNM, LVI) [9,10], lung cancer (T stage, pleural invasion, LNM, advance stage disease, LVI) [11], head and neck cancer (LNM, LVI, and PNI) [12,13], and breast cancer (T stage, LNM, LVI) [14,15]. The prognostic significance of TB has been explored in patients with gynecologic cancer, especially uterine cervical squamous cell carcinoma [16,17,18,19,20] and endometrial adenocarcinoma [21,22,23]. However, the significance of TB in survival and the criteria for assessing this method have not been confirmed in gynecologic cancers.

Thus, the main objective of the present study was to confirm the prognostic significance of TB in gynecological cancers. In addition, we aimed to compare the three assessment systems that have been used previously. Furthermore, we investigated the relationship between TB and EMT in gynecologic cancers.

## 2. Materials and Methods

### 2.1. Search Strategy

This meta-analysis study was registered in PROSPERO (CRD42021251435) and approved by the Institutional Review Board of the Catholic University of Korea College of Medicine (UC21ZISI0059). Three major electronic databases, Medline, EMBASE, and the Cochrane Library, were searched for relevant English-written articles published through April 2021. The search terminologies are summarized in Table S1. Next, the references were manually searched by cross-referencing key articles. EndNote X20 (Build 10136, Thomson Reuters, New York, NY, USA) was used to manage the database.

### 2.2. Inclusion and Exclusion Criteria

In this meta-analysis, the following inclusion criteria were used: (1) the relationship between TB and the survival rates of patients was assessed; (2) TB was diagnosed accurately by precise histopathologic examination; (3) studies provided enough information about the hazard ratio (HR) of patient survival; (4) studies showed an association between TB and clinicopathological features using at least two parameters; and (5) the articles were written in English. The following were excluded: (1) duplicate studies, reviews, case reports, letters, and conference proceedings; (2) studies that did not show an association between TB and survival or clinicopathological parameters; (3) studies concerned with cancer cell lines and animal models; and (4) studies with insufficient data related to HRs and 95% confidence intervals (CIs) that could not be extracted or calculated.

### 2.3. Data Extraction and Assessment of Study Quality

Independent reviewers (M.J.A. and K.Y.) extracted the data; any disagreements during the process were resolved by consensus involving three reviewers (M.J.A., K.Y. and Y.C.). The following data were extracted from all studies: author/year, country, ethnicity, cell type, age (year, median age), patients, sampling year, pathological stage, follow-up, treatment, staining, cut-off value, field of view, overall survival (OS), disease-free survival (DFS), and pathologist involvement. The Quality In Prognosis Studies (QUIPS) tool was used to assess the risk of bias and select studies that qualified for the analysis.

### 2.4. Statistical Analysis

Statistical analysis was performed using Review Manager software (version 5.3; Cochrane Collaboration, Copenhagen, Denmark). Pooled HRs with 95% CIs were used to evaluate the association between TB and survival. HR values greater than one indicated poor survival and vice versa. The association between TB and other clinicopathological parameters was calculated using the Mantel–Haenszel pooled odds ratio with 95% CI and the combined effective value. An I^2^ value of less than 50% indicated no heterogeneity among the studies. Subgroup analysis was performed to investigate the heterogeneity.

## 3. Results

### 3.1. Eligible Studies

**Figure 1:**
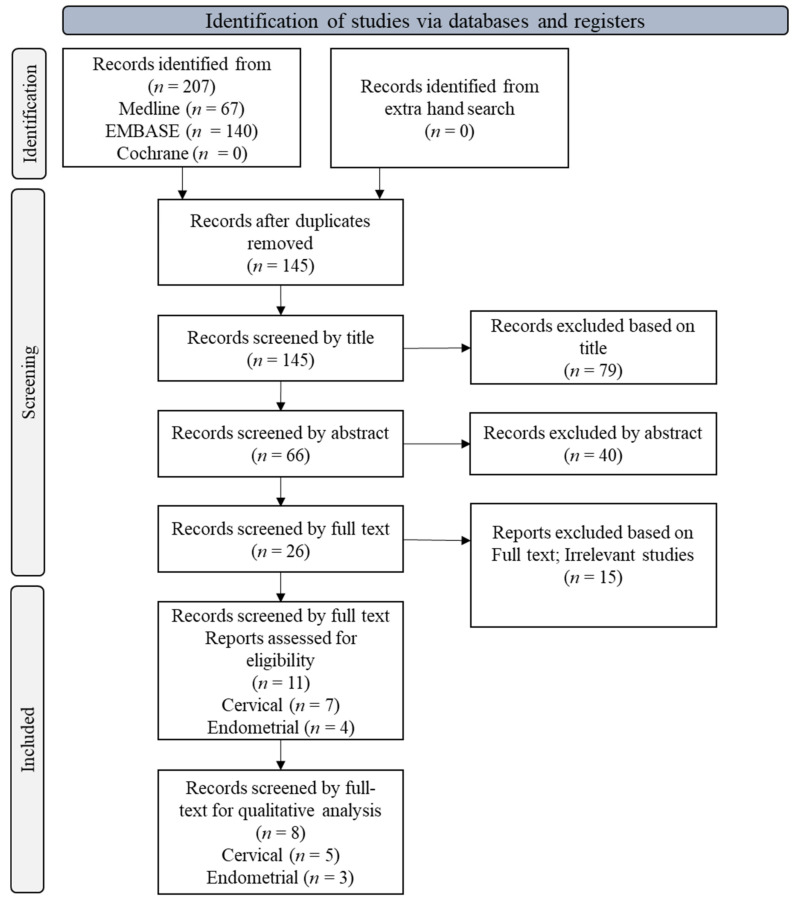
PRISMA flow diagram showing the study selection process.

The preliminary pool of selected literature included 207 articles from Medline, EMBASE, and Cochrane Library (Figure 1). After 62 duplicate articles were removed, 145 records were screened using the reference type. Only eleven articles (seven uterine cervical and four endometrial cancers) fulfilled the inclusion criteria for this meta-analysis based on data related to prognosis, clinicopathological parameters, assessment method, and association of EMT with TB. Eight studies (five cervical and three endometrial cancers) were used for the qualitative analysis (Figure 1). Almost all studies showed a low risk of bias (Figure S1).

### 3.2. Study Characteristics

Finally, eleven studies were selected for analysis. The studies were conducted in nine countries and were published between 2012 and 2020 (Table 1 and Table S2). Among them, two studies used a novel method that combined TB and tumor nest size; therefore, we could not determine the HRs of TB. Thus, we calculated survival rates according to TB from the data [18,20]. The total number of patients was 2009, ranging from 91 to 834, and diagnoses included stages I–IV (Table 1 and Table S2).

### 3.3. High-Grade Tumor Budding and Prognosis in Gynecological Cancer Patients

**Figure 2:**
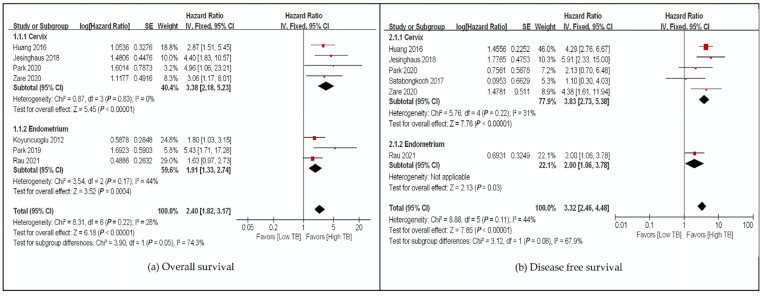
Subgroup hazard ratios analyzing the tumor budding expression, (**a**) overall survival, (**b**) disease-free survival in gynecological cancer patients by organs. ■ The location of square epresents the hazard atio and the size means individual effect of studies. 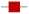 The black line represents 95% confidence iterval of the study. ◆ The diamond represents pooled hazard ratio and its edge shows 95% confidence interval.

**Figure 3:**
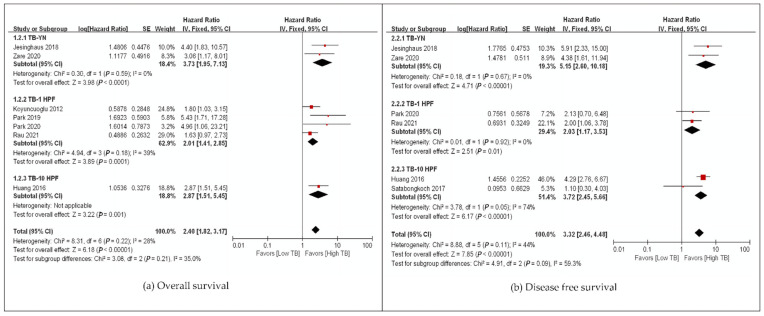
Subgroup hazard ratios analyzing the tumor budding expression, (**a**) overall survival, (**b**) disease-free survival in gynecological cancer patients by assessment method. ■ The location of square epresents the hazard atio and the size means individual effect of studies. 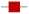 The black line represents 95% confidence iterval of the study. ◆ The diamond represents pooled hazard ratio and its edge shows 95% confidence interval.

We evaluated the correlation between TB and OS among 1652 gynecological patients from eight studies (Table 1 and Table S2). The pooled HR for OS and DFS demonstrated that high-grade TB was significantly associated with poor OS (HR: 2.40, 95% CI: 1.82–3.17, *p* < 0.00001) and DFS (HR: 3.32, 95% CI: 2.46–4.48, *p* < 0.00001), regardless of the cancer origin site with low heterogeneity (Figure 2). Only studies on uterine cervical and endometrial carcinomas were included in this analysis (Figure 2). One study on ovarian clear cell carcinoma was identified during the search in the present study, but it was a conference presentation paper, and we could not find data available for meta-analysis [27]. High-grade TB was a poor survival marker for every subgroup analysis based on assessment methods, ethnicity, and univariate and multivariate analyses with low heterogeneity (Figure 3, Figures S2 and S3). In addition, one study analyzed distant metastasis-free survival (DMFS), and high-grade TB was a marker of poor survival [25] (Table 1). Meanwhile, one study performed by Cao et al. [25] assessed DMFS and showed that TB was positively correlated with distant metastasis (*p* = 0.012).

### 3.4. High-Grade Tumor Budding and Clinicopathological Parameters

The main clinicopathological parameters based on TB from all studies included in the meta-analysis are summarized in Table 2 and Table S3. High-grade TB was significantly associated with clinicopathological parameters such as, age, stage (III and IV), depth of invasion (more than half), N stage (N1, N2, N3), M stage (MI), grade (G3), lymphovascular invasion (present), and perineural invasion (Table 3, Figure S4, Table S3).

### 3.5. Tumor-Budding Assessment Methods: Present/Absent (TB-YN), Maximum of One High Power Field (TB-1HPF), and a Total of 10 High-Power Fields (TB-10HPF)

**Figure 4:**
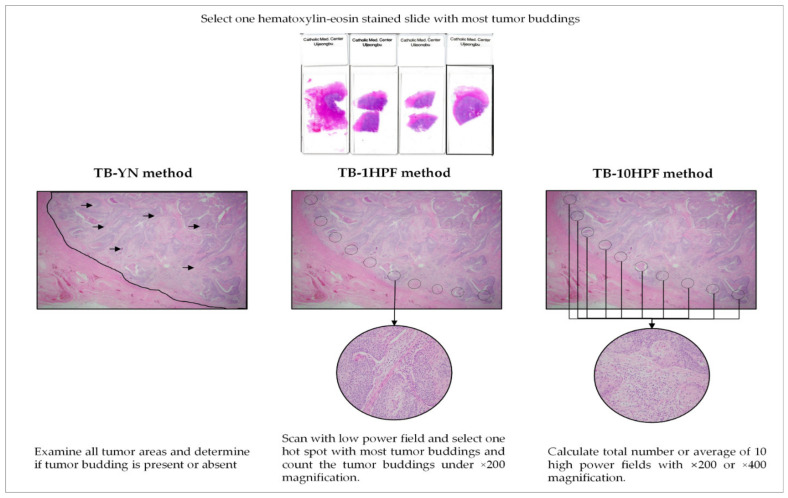
Description of three representative tumor budding assessment method.

Three assessment methods were used to evaluate TB (Figure 4). First, using the TB-YN method, most studies classified tumors based on the presence or absence of TB [18,20,24]. Secondly, for the TB-1HPF method, researchers searched whole slides, selected one “hotspot” under 200× magnification, and counted the number of TBs [19,21,22,23]. Rau et al. [24] used the International Tumor Budding Consensus Conference (ITBCC) method. Using this method, the same number of TBs as the TB-1HPF method was determined, but the counted TBs were adjusted to fit an area of 0.785 mm^2^ [5]. Finally, using the TB-10HPF method, the total number or the average number of buds in 10 HPF at 200× or 400× magnification was obtained [16,17,18,20,24,26] (Table 1).

### 3.6. Tumor Budding and Molecular Features

Four studies investigated the relationship between TB and EMT markers, tumor microenvironmental factors, hormone receptors, and molecular subclassifications of cancer. The detailed findings of each study are summarized in Table 2. For all EMT studies, we found decreased expression levels of E-cadherin, estrogen receptor (ER), progesterone receptor (PR), and aberrant expression of β-catenin (Table 3). In addition, microvascular density was significantly related to TB (R = 0.3, *p* = 0.0002) (Table 3). The role of TB in survival stratification was best for the non-specific molecular profile (NSMP) group, followed by the mismatch repair deficiency (MMRd) group (Table 3).

## 4. Discussion

We confirmed that TB was a poor prognostic marker and aggressive clinicopathological factor for gynecological cancers (Table 1 and Figures S3 and S4). Patients with high-grade TB showed poor survival in all subgroup analyses according to ethnicity, univariate vs. multivariate analysis, and assessment methods. In addition, TB was an independent predictor of prognosis for the NSMP and MMRd groups in endometrial cancers [23] (Table 3). To the best of our knowledge, this is the first comprehensive systematic review and meta-analysis to evaluate the correlation between TB and gynecological cancer.

For the present review, we aimed to identify all studies on TB in gynecological cancer (Table 1, Table S1). The low inclusion rate (3.84%) of papers after the screening of the literature was due to the rigorous process used to find all papers related to TB in gynecological cancer. Despite our efforts, we could not find studies on vulvar, vaginal, or fallopian tubal cancers. We found only seven studies on cervical cancer [16,17,18,19,20,24,25], four on endometrial cancer [21,22,23,26], and one on ovarian cancer [27], which suggests that TB had no prognostic significance in 69 patients with clear cell carcinoma. However, that study was a poster presentation, and no data were available for meta-analysis; the authors only suggested that TB had no prognostic significance [27]. TB may or may not be a poor survival factor for ovarian cancer because the tumor-spreading pathway of ovarian cancer is different from that of other solid cancers, in that it involves direct seeding into the body cavity [28]. Further studies on the prognostic significance of TB in ovarian cancer are required.

Furthermore, three assessment methods (TB-YN, TB-1HPF, and TB-10HPF) were used to interpret TB (Figure 4). Owing to its simplicity, TB-YN showed the highest reproducibility (I^2^ = 0%) among all three methods. However, its accuracy is questionable because TB-mimicking macrophages, tangentially sectioned glands, and/or apoptotic tumor cells can cause diagnostic errors [9,18,20]. TB was associated with poor OS and DFS regardless of the assessment method; however, standardization or consensus meetings for TB assessment for gynecologic cancers are still needed.

In addition, the ITBCC is a popular scoring system used by pathologists that was approved in 2016 to create a standardized scoring system for colorectal cancer [5]. TBs were counted under 200× magnification in an HPF of hot spots and recalculated for a field area of 0.785 mm^2^ [5]. The ITBCC was developed for colorectal carcinoma; however, pathologists use it for other cancers. Rau et al. also applied the ITBCC method to endometrial cancers and found that it was an independent prognostic factor for DFS (HR = 2.0, 95% CI = 1.10–3.80, *p* = 0.0329) [23]. This scoring system, which is affiliated with the reputed committee, is still required for the standardization of gynecological cancer.

In addition, hematoxylin and eosin (HE) or immunohistochemistry (IHC) staining with pancytokeratin antibody is preferable for TB scoring. The significant advantage of IHC was that it showed a small area of TB more clearly, especially in the inflammatory background, and reduced subjectivity during the examination of slides [5,29]. However, it also stained apoptotic tumor cells and other cell-related debris, which should not be counted in the final number. Moreover, emerging cancer studies have shown no significant difference between HE and IHC (R = 0.92, *p* < 0.001) [5,30]. The ITBCC suggests that HE is preferred for routine diagnosis because of its lower price, while pancytokeratin IHC should be exploited for complicated cases [5]. In our analysis, we found two studies that used IHC-based antibodies with different clones, while the rest of the studies exploited HE staining. Although all these studies showed a poor prognosis of TB, further studies that directly compare the results of HE and IHC are still needed.

In endometrial cancers, as with other solid tumors, it is possible that high-grade TB is associated with EMT [8,11,29,31,32,33]. Decreased levels of E-cadherin, ER, PR, and aberrant β-catenin expressions were associated with high-grade TB in endometrial cancers [22,26] (Table 3). E-cadherin is a cell-adhesive molecule, and the loss of expression causes tumor cell dissociation, which is the first step of EMT [4]. A reduction in the amount of β-catenin at the cell membrane and/or inside the cytoplasm often leads to loss of E-cadherin [4,33]. ER and PR inhibit EMT by increasing signaling transduction cascades such as TGF-β, WNT/β-catenin, and NF-κB [34,35]. In addition, expression of ER and PR is usually associated with successful treatment with medroxyprogesterone acetate in endometrial cancers, therefore their loss is associated with a more invasive phenotype and chemoresistance [36].

Angiogenesis plays an important role in the growth and progression of cancer [37]. Thus, antiangiogenic agents have been introduced in various solid cancers, including gynecologic cancers [38]. Kluz et al. [26] showed that TB is related to angiogenesis in endometrial cancer, and it therefore may be a predictor of the response to these target agents. 

Our study has a few limitations: (i) studies not published in the English language were excluded due to the challenges of obtaining the precise data, which may bias our results; (ii) for studies without HRs with 95% CIs, the data were extracted using an indirect method before the pooled HR was calculated, which may have compromised the accuracy of the data. (iii) Few studies on the relationship of TB and EMT in gynecologic cancers have been conducted; therefore, further research is required to confirm the association between TB and EMT. Regardless of the above limitations, our meta-analysis revealed the prognostic and clinicopathological significance of TB in gynecological cancers.

## 5. Conclusions

We conclude that high-grade TB is significantly associated with poor prognosis, regardless of histologic type, ethnicity, and assessment method. The standardization of TB assessment methods through large consensus meetings is still needed. Moreover, high-grade TB may be associated with EMT, similar to other solid cancers. We believe that assessments of TB should be routinely performed when pathological diagnoses are reported.

## Figures and Tables

**Table 1:** Main characteristics of all gynecological cancer studies included in the meta-analysis.

Site	Authors Year	Histology	Patients (*n*)	Staining Method	Assessment Method	Field of View	Cutoff	Outcome
Cervix	Huang et al., 2016 [16]	SCC	834	Pancytokeratin (AE1/AE3)	TB-10HPF	×200	≥5	OS, DFS
	Satabongkoch et al., 2017 [17]	ADC	129	HE	TB-10HPF	×400	≥15	DFS
	Jesinghaus et al., 2018 [18]	SCC	125	HE	TB-YN TB-10HPF	×200	>0 ≥15	OS *, DFS *
	Park et al., 2020 [19]	SCC, ADC	136	HE	TB-1HPF	×200	≥5	OS, DFS
	Stanulović et al., 2020 [24]	SCC, ADC	91	HE	TB-YN TB-10HPF	×200	>0 ≥15	-
	Zare et al., 2020 [20]	SCC	94	HE	TB-YN TB-10HPF	×200	>0 ≥15	OS *, DFS *
	Cao et al., 2020 [25]	SCC	122	HE Pancytokeratin (CD31, CD34))	TB-10HPF	×200	>0 ≥15	-
Endometrium	Koyuncuoglu et al., 2012 [21]	-	112	HE Pancytokeratin (C11) CD34 **	TB-1 HPF	×200	≥5	OS
	Park et al., 2019 [22]	-	96	HE	TB-1 HPF	×200	≥5	OS
	Kluz et al., 2020 [26]	-	137	Laminin 5γ2	TB-10 HP	×200	≥5	-
	Rau, T.T. et al., 2020 [23]	-	255	HE	TB-1 HPF	×200 (0.785 mm^2^)	≥5	OS, DFS

Abbreviations: SCC: Squamous cell carcinoma, OS: Overall survival, DFS: Disease-free survival, ADC: Adeno cell carcinoma, HE: Hematoxylin and eosin stain, TB-YN: Tumor budding absent/present, TB-1 HPF Tumor budding at 1 high power field, TB-10 HPF: Tumor budding at 10 high power field. * These data are indirectly extracted by calculating the odd ratio from the sample size. ** CD34 is used for excluding tumor emboli.

**Table 2:** Summary of a meta-analysis evaluating the relationship of tumor budding with clinicopathological parameters of gynecological cancer.

Parameters	Number of Studies	Number of Patients	Pooled OR (95% CI)	*p*-Value	Heterogeneity		
					I^2^ (%)	*p*-Value	Model
Age	3	345	1.77 [0.97, 3.20]	0.06	65	0.06	Fixed
Stage (III and IV)	7	1361	1.94 [1.41, 2.66]	<0.001	19	0.28	Fixed
Depth of invasion (over than half)	2	225	2.68 [1.33, 5.40]	0.006	71	0.06	Fixed
N stage (N1, N2, N3)	6	1145	4.05 [2.93, 5.60]	<0.001	0%	0.59	Fixed
M stage (M1)	2	732	4.60 [1.67, 12.67]	0.003	0%	0.85	Fixed
Grade (G3)	7	1309	2.26 [1.70, 2.99]	<0.001	60%	0.02	Fixed
Lymphovascular invasion (present)	7	1270	4.18 [3.09, 5.66]	<0.001	71%	0.002	Fixed
Perineural invasion (present)	4	950	2.25 [1.31, 3.88]	0.004	23%	0.04	Fixed

**Table 3:** Summary of tumor budding’s relationship with molecular markers.

Site	References	EMT, Immune Cell and Other Markers	Main Findings
Endometrium	Koyuncuoglu et al., 2012 [21]	E-cadherin	Tumor budding adversely correlated with the presence of E-cadherin expression, but this relation was not statistically significant (*p* = 0.359).
	Park et al., 2019 [22]	ER, PR, p53, E-cadherin, β-catenin	Tumor budding was associated with reduced expression of hormone receptors (ER and PR) and aberrant β-catenin expression combined with loss of E-cadherin expression. (*p* = 0.002, *p* = 0.836). The expression of p53 was wild type in all cases.
	Rau, T.T. et al., 2020 [23]	Molecular classification (POLEmut, MMRd, NSMP, and p53abn)	Survival stratification of tumor budding was best in the NSMP group followed by the MMRd group.
	Kluz et al., 2020 [26]	CD34	Microvascular density is statistically significant with tumor budding (R = 0.3, *p* = 0.0002)

Abbrevation: MMRd: Mismatch repair deficient, NSMP: Non-specific mutation profile.

## Data Availability

The data presented in this study are available upon reasonable request from the corresponding author.

## Supplementary Materials

The following supporting information can be downloaded at: https://www.mdpi.com/article/10.3390/cancers14061431/s1, Figure S1: Risk of the bias QUIPS tool; Figure S2: Subgroup analysis of tumor budding according to the univariate vs. multivariate analysis in gynecological cancer patients; overall survival (a) disease-free survival (b); Figure S3: Subgroup analysis of tumor budding according to ethnicity in gynecological cancer patients; overall survival (a) disease-free survival (b); Figure S4: Subgroup hazard ratio analysis of tumor budding and pathological parameters in gynecological cancer patients by (a) age (b) stage (c) depth of invasion (d) N stage (e) M stage (f) tumor grade (g) lymphovascular invasion (h) perineural invasion,; Table S1: Detailed overview of keywords used for search strategy; Table S2: Main characteristics of all included gynecological cancer studies; Table S3: Characteristics of patients’ clinicopathological data included in the meta-analysis.

## References

[1] Arbyn M., Weiderpass E., Bruni L., de Sanjose S., Saraiya M., Ferlay J., Bray F. (2020). Estimates of incidence and mortality of cervical cancer in 2018: A worldwide analysis. Lancet Glob. Health.

[2] Vale C.L., Tierney J., Bull S.J., Symonds P.R. (2012). Chemotherapy for advanced, recurrent or metastatic endometrial carcinoma. Cochrane Database Syst. Rev..

[3] Ledford L.R., Lockwood S. (2019). Scope and Epidemiology of Gynecologic Cancers: An Overview. Semin. Oncol. Nurs..

[4] Lugli A., Zlobec I., Berger M.D., Kirsch R., Nagtegaal I.D. (2021). Tumour budding in solid cancers. Nat. Rev. Clin. Oncol..

[5] Lugli A., Kirsch R., Ajioka Y., Bosman F., Cathomas G., Dawson H., El Zimaity H., Flejou J.F., Hansen T.P., Hartmann A. (2017). Recommendations for reporting tumor budding in colorectal cancer based on the International Tumor Budding Consensus Conference (ITBCC) 2016. Mod. Pathol..

[6] Yim K., Won D.D., Lee I.K., Oh S.T., Jung E.S., Lee S.H. (2017). Novel predictors for lymph node metastasis in submucosal invasive colorectal carcinoma. World J. Gastroenterol..

[7] Rogers A.C., Winter D.C., Heeney A., Gibbons D., Lugli A., Puppa G., Sheahan K. (2016). Systematic review and meta-analysis of the impact of tumour budding in colorectal cancer. Br. J. Cancer.

[8] Lugli A., Karamitopoulou E., Zlobec I. (2012). Tumour budding: A promising parameter in colorectal cancer. Br. J. Cancer.

[9] Yim K., Jang W.M., Lee S.H. (2021). Modified Tumor Budding as a Better Predictor of Lymph Node Metastasis in Early Gastric Cancer: Possible Real-World Applications. Cancers.

[10] Guo Y.X., Zhang Z.Z., Zhao G., Zhao E.H. (2019). Prognostic and pathological impact of tumor budding in gastric cancer: A systematic review and meta-analysis. World J. Gastrointest. Oncol..

[11] Yamaguchi Y., Ishii G., Kojima M., Yoh K., Otsuka H., Otaki Y., Aokage K., Yanagi S., Nagai K., Nishiwaki Y. (2010). Histopathologic Features of the Tumor Budding in Adenocarcinoma of the Lung Tumor Budding As an Index to Predict the Potential Aggressiveness. J. Thorac. Oncol..

[12] Zhu Y., Liu H., Xie N., Liu X., Huang H., Wang C., Hou J. (2019). Impact of tumor budding in head and neck squamous cell carcinoma: A meta-analysis. Head Neck.

[13] Karjol U., Jonnada P., Annavarjula V., Cherukuru S., Chandranath A., Anwar A. (2020). Prognostic Role of Tumor Budding in Carcinoma Tongue: A Systemic Review and Meta-Analysis. Cureus.

[14] Liang F.L., Cao W., Wang Y.L., Li L.R., Zhang G.J., Wang Z. (2013). The prognostic value of tumor budding in invasive breast cancer. Pathol. Res. Pract..

[15] Lloyd A.J., Ryan E.J., Boland M.R., Elwahab S.A., Malone C., Sweeney K.J., Barry K.M., McLaughlin R., Kerin M.J., Lowery A.J. (2020). The histopathological and molecular features of breast carcinoma with tumour budding—A systematic review and meta-analysis. Breast Cancer Res. Treat..

[16] Huang B., Cai J., Xu X., Guo S., Wang Z. (2016). High-Grade Tumor Budding Stratifies Early-Stage Cervical Cancer with Recurrence Risk. PLoS ONE.

[17] Satabongkoch N., Khunamornpong S., Pongsuvareeyakul T., Settakorn J., Sukpan K., Soongkhaw A., Intaraphet S., Suprasert P., Siriaunkgul S. (2017). Prognostic Value of Tumor Budding in Early-Stage Cervical Adenocarcinomas. Asian Pac. J. Cancer Prev..

[18] Jesinghaus M., Strehl J., Boxberg M., Brühl F., Wenzel A., Konukiewitz B., Schlitter A.M., Steiger K., Warth A., Schnelzer A. (2018). Introducing a novel highly prognostic grading scheme based on tumour budding and cell nest size for squamous cell carcinoma of the uterine cervix. J. Pathol. Clin. Res..

[19] Park J.Y., Chong G.O., Park J.Y., Chung D., Lee Y.H., Lee H.J., Hong D.G., Han H.S., Lee Y.S. (2020). Tumor budding in cervical cancer as a prognostic factor and its possible role as an additional intermediate-risk factor. Gynecol. Oncol..

[20] Zare S.Y., Aisagbonhi O., Hasteh F., Fadare O. (2020). Independent Validation of Tumor Budding Activity and Cell Nest Size as Determinants of Patient Outcome in Squamous Cell Carcinoma of the Uterine Cervix. Am. J. Surg. Pathol..

[21] Koyuncuoglu M., Okyay E., Saatli B., Olgan S., Akin M., Saygili U. (2012). Tumor budding and E-Cadherin expression in endometrial carcinoma: Are they prognostic factors in endometrial cancer?. Gynecol. Oncol..

[22] Park J.Y., Hong D.G., Chong G.O., Park J.Y. (2019). Tumor Budding is a Valuable Diagnostic Parameter in Prediction of Disease Progression of Endometrial Endometrioid Carcinoma. Pathol. Oncol. Res..

[23] Rau T.T., Bettschen E., Büchi C., Christe L., Rohner A., Müller M.D., Carlson J.W., Imboden S., Zlobec I. (2021). Prognostic impact of tumor budding in endometrial carcinoma within distinct molecular subgroups. Mod. Pathol..

[24] Stanulović N., Kapicl T.I., Mandić A., Gutić B. (2020). Tumor budding in cervical carcinoma: Associations with some clinical and pathological factors. Arch. Oncol..

[25] Cao L., Sun P.L., He Y., Yao M., Gao H. (2020). Desmoplastic Reaction and Tumor Budding in Cervical Squamous Cell Carcinoma are Prognostic Factors for Distant Metastasis: A Retrospective Study. Cancer Manag. Res..

[26] Kluz T., Łoziński T., Czekierdowska S., Stachowicz N., Gurynowicz G., Chróściel M., Czekierdowski A. (2020). Tumor budding index and microvessel density assessment in patients with endometrial cancer: A pilot study. Oncol. Lett..

[27] Lin L., Zamuco R., Shukla P. (2021). Small tumor nests are associated with poor clinical outcome in clear cell carcinoma of ovary. Lab. Investig..

[28] Kumar V., Abbas A., Aster J., Turner J.R. (2020). Robinns & Cotran, Pathologic Basis of Diseases.

[29] Kazama S., Watanabe T., Ajioka Y., Kanazawa T., Nagawa H. (2006). Tumour budding at the deepest invasive margin correlates with lymph node metastasis in submucosal colorectal cancer detected by anticytokeratin antibody CAM5.2. Br. J. Cancer.

[30] van Wyk H.C., Park J., Roxburgh C., Horgan P., Foulis A., McMillan D.C. (2015). The role of tumour budding in predicting survival in patients with primary operable colorectal cancer: A systematic review. Cancer Treat. Rev..

[31] Wang C., Huang H.Z., Huang Z.Q., Wang A.X., Chen X.H., Huang L., Zhou X.F., Liu X.Q. (2011). Tumor budding correlates with poor prognosis and epithelial-mesenchymal transition in tongue squamous cell carcinoma. J. Oral. Pathol. Med..

[32] Gujam F.J.A., McMillan D.C., Mohammed Z.M.A., Edwards J., Going J.J. (2015). The relationship between tumour budding, the tumour microenvironment and survival in patients with invasive ductal breast cancer. Br. J. Cancer.

[33] Grigore A.D., Jolly M.K., Jia D.Y., Farach-Carson M.C., Levine H. (2016). Tumor Budding: The Name is EMT. Partial EMT. J. Clin. Med..

[34] Guttilla I.K., Adams B.D., White B.A. (2012). ERalpha, microRNAs, and the epithelial-mesenchymal transition in breast cancer. Trends Endocrinol. Metab..

[35] Van der Horst P.H., Wang Y.Y., Vandenput I., Kuhne L.C., Ewing P.C., van IJcken W.F.J., van der Zee M., Amant F., Burger C.W., Blok L.J. (2012). Progesterone Inhibits Epithelial-to-Mesenchymal Transition in Endometrial Cancer. PLoS ONE.

[36] Wei J., Zhang W., Feng L., Gao W. (2017). Comparison of fertility-sparing treatments in patients with early endometrial cancer and atypical complex hyperplasia: A meta-analysis and systematic review. Medicine.

[37] Mazurek A., Kuc P. (2005). Angiogenesis-prognostic factor in patients with endometrial cancer. Ginekol. Pol..

[38] Maj E., Papiernik D., Wietrzyk J. (2016). Antiangiogenic cancer treatment: The great discovery and greater complexity (Review). Int. J. Oncol..

