# Supplementary material for: Tumor Budding in Gynecologic Cancer as a Marker for Poor Survival: A Systematic Review and Meta-Analysis of the Perspectives of Epithelial–Mesenchymal Transition
Source: Cancers (Basel). 2022 Mar 10;14(6):1431. doi: 10.3390/cancers14061431 (PMC8946491; doi:10.3390/cancers14061431)
Supplement: Supplementary file 1 [file cancers-14-01431-s001.zip › cancers-1595555-supplementary.pdf]

Supplementary Materials: Tumor Budding in Gynecologic Cancer as a Marker for Poor Survival: A Systematic Review and Meta-Analysis in the Perspective of Epithelial-Mesenchymal Transition

Muhammad Joan Ailia, Nishant Thakur, Yosep Chong and Kwangil Yim

	Zare 2020	Stanulović 2020	Satabongkoch 2017	Rau 2021	Park 2020	Park 2019	L. Cao 2020	Koyuncuoglu 2012	Kluz 2020	Jesinghuas 2018	Huang 2016
Study participation	+	+	+	+	+	+	+	+	+	+	+
Study attrition	+	+	+	+	+	+	+	+	+	+	+
Prognostic factor measurement	+	+	+	+	+	+	?	+	+	+	+
Outcome measurement	+	+	+	+	+	+	+	+	+	+	+
Study Confounding	+	?	+	?	+	+	?	+	+	+	+
Statistical analysis and reporting	+	+	+	+	+	+	+	+	+	+	+

Figure S1. Risk of the bias Quality In Prognosis Studies (QUIPS) tool

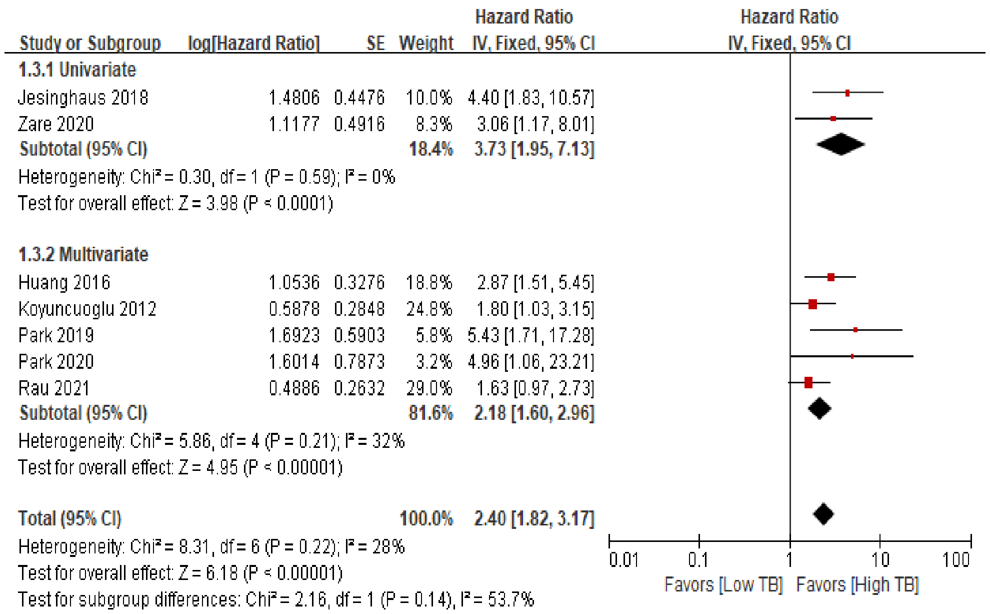

(a) Overall survival

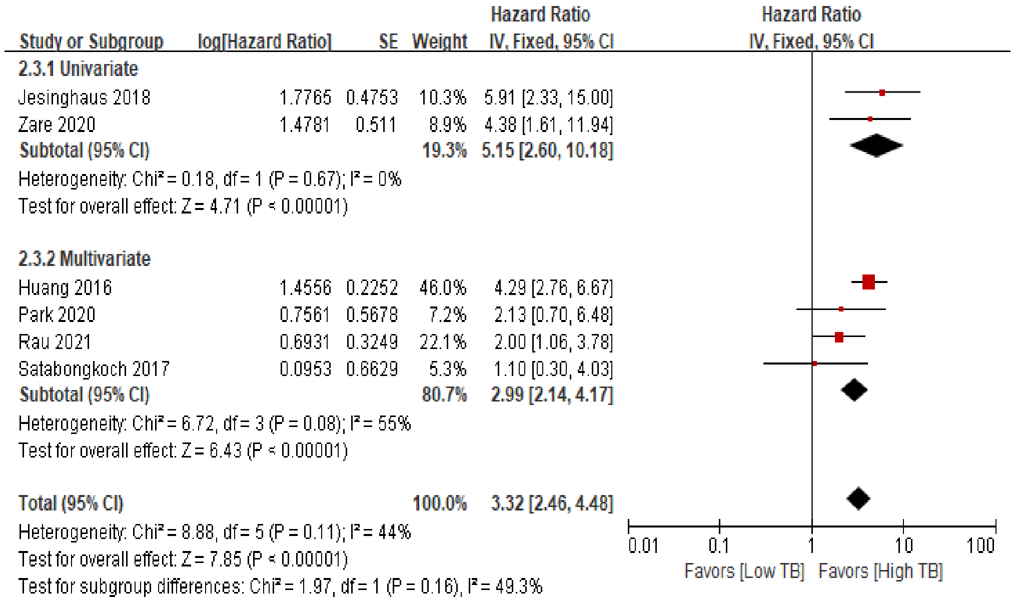

(b) Disease free survival

Figure S2. Subgroup analysis of tumor budding according to the univariate vs multivariate analysis in gynecological cancer patients; overall survival (**a**) disease free survival (**b**).

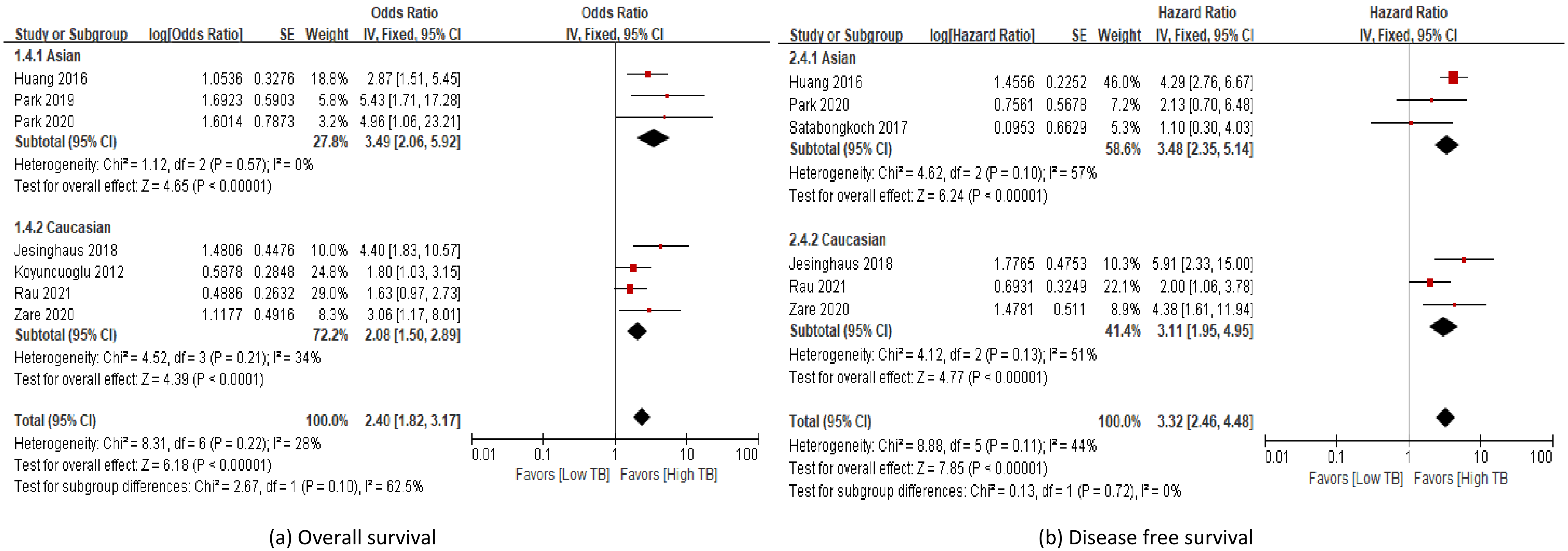

Figure S3. Subgroup analysis of tumor budding according to the ethnicity in gynecological cancer patients; overall survival (**a**) disease free survival (**b**).

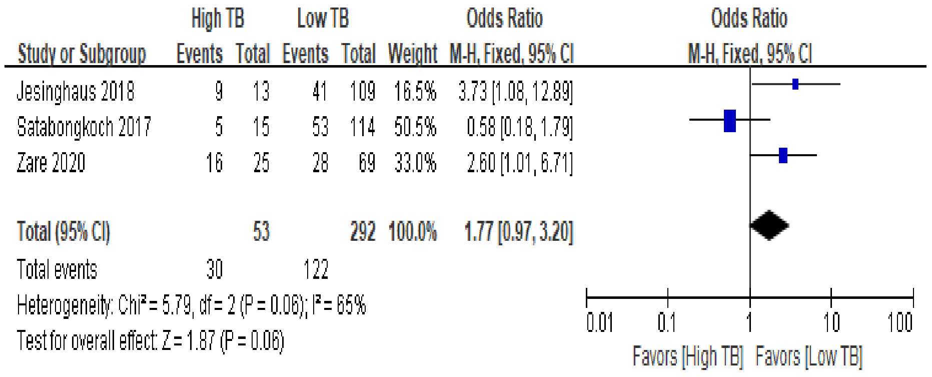

(a) Age

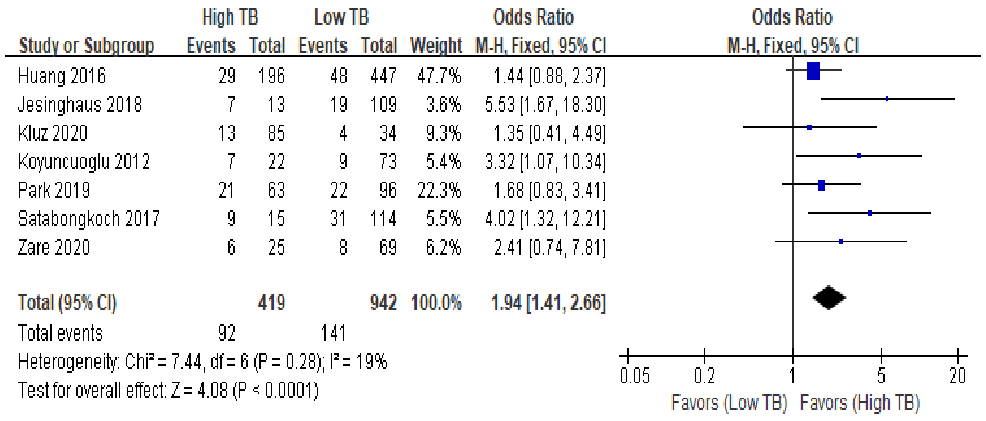

(b) Stage

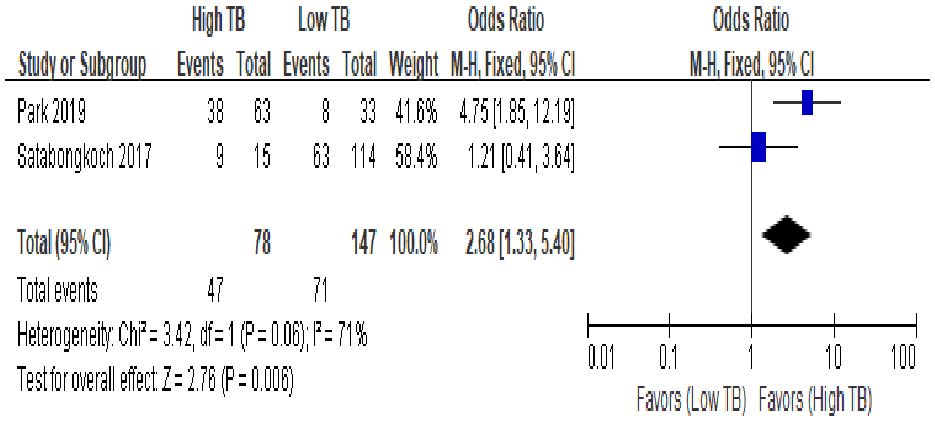

(c) Depth of invasion

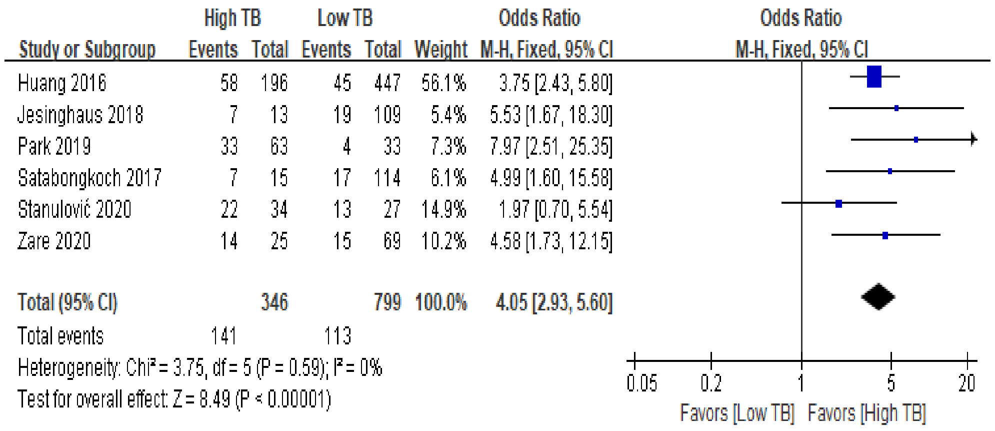

(d) N stage

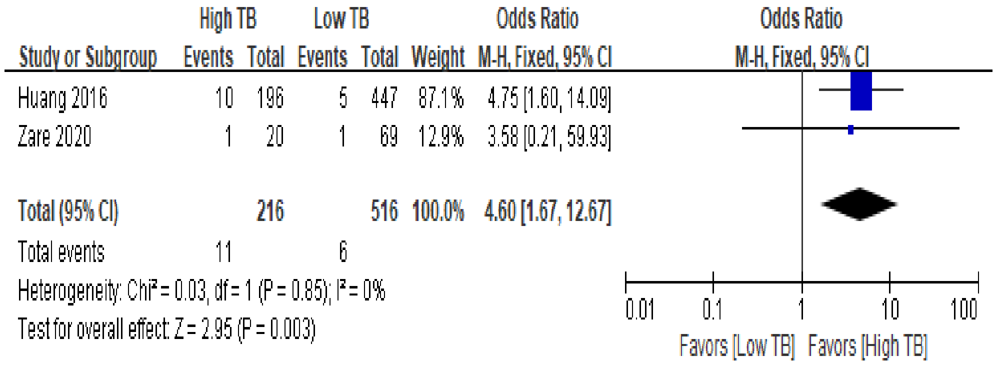

(e) M Stage

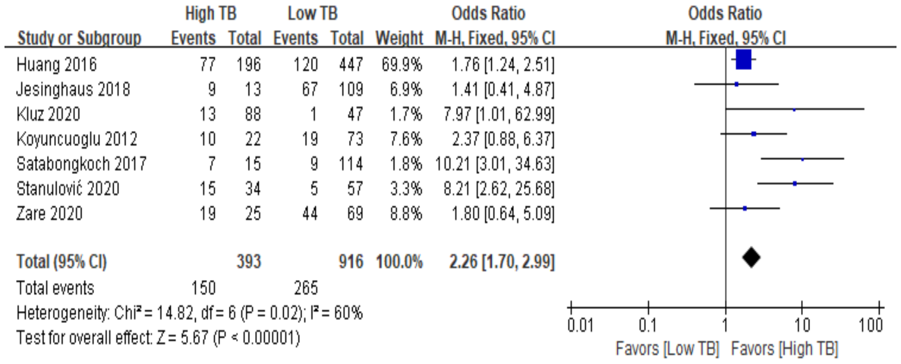

(f) Tumor grade

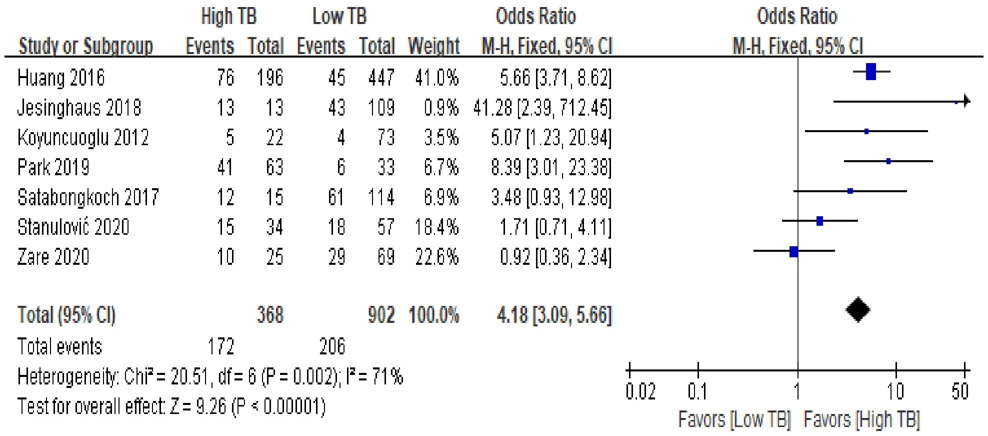

(g) Lymphovascular invasion

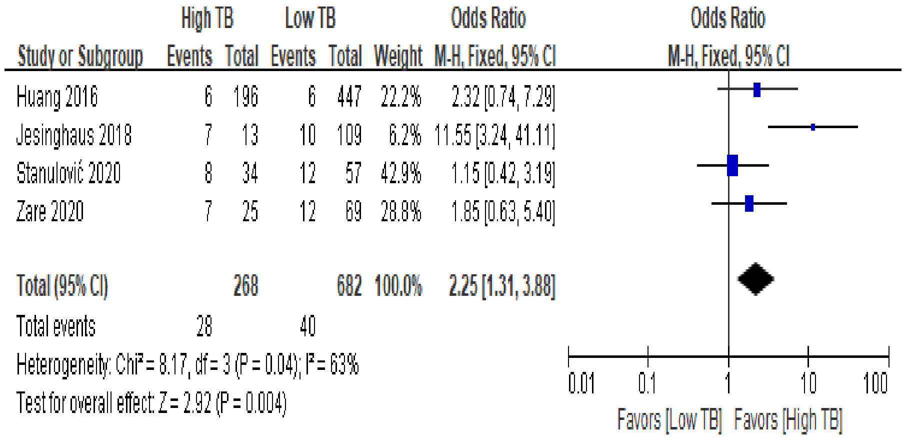

(h) Perineural invasion

Figure S4. Subgroup hazard ratio analyzing the tumor budding expression and pathological parameters in gynecological cancer patients (**a**) age (**b**) stage (**c**) depth of invasion (**d**) N stage (e) M stage (**f**) tumor grade (**g**) lymphovascular invasion (**h**) perineural invasion.

Supplementary Table S1. Detailed overview of keywords used for search strategy

Data base	Search	Keywords
Medline Library	#1	"Gynecologic Oncology"[TW] OR "Gynecologic Cancer"[TW]
	#2	"Ovarian Neoplasms"[Mesh]
	#3 Combine	#1 OR #2
	#4	"Endometrial Neoplasms"[Mesh]
	#5	"Endometrial Neoplasms"[TW] OR "Endometrial Neoplasm"[TW] OR "Neoplasm, Endometrial"[TW] OR "Neoplasms, Endometrial"[TW] OR "Endometrial Carcinoma"[TW] OR "Carcinoma, Endometrial"[TW] OR "Carcinomas, Endometrial"[TW] OR "Endometrial Carcinomas"[TW] OR "Endometrial Cancer"[TW] OR "Cancer, Endometrial"[TW] OR "Cancers, Endometrial"[TW] OR "Endometrial Cancers"[TW] OR "Endometrium Cancer"[TW] OR "Cancer, Endometrium"[TW] OR "Cancers, Endometrium"[TW] OR "Cancer of the Endometrium"[TW] OR "Carcinoma of Endometrium"[TW] OR "Endometrium Carcinoma"[TW] OR "Endometrium Carcinomas"[TW] OR "Cancer of Endometrium"[TW] OR "Endometrium Cancers"[TW]
	#6	"Uterine Cervical Neoplasms"[Mesh]
	#7 Combine	#5 OR #6
	#8 Combine	"Carcinoma, Endometrioid"[Mesh]
	#9 Combine	"Carcinoma, Endometrioid"[TW] OR "Carcinomas, Endometrioid"[TW] OR "Endometrioid Carcinoma"[TW] OR "Endometrioid Carcinomas"[TW] OR "Adenocarcinoma, Endometrioid"[TW] OR "Adenocarcinomas, Endometrioid"[TW] OR "Endometrioid Adenocarcinomas"[TW] OR "Endometrioid Adenocarcinoma"[TW] OR "Endometrial Endometrioid Carcinoma"[TW]
	#10 Combine	#8 AND #9
	#11 Combine	"tumor budding"[TW] OR "budding"[TW] OR "High-Grade Tumor Budding"[TW] OR "tumor-cell dissociation"[TW]
	#12 Combine	#10 AND #11

Data base	Search	Keywords
EMBASE Library	#1	"Gynecologic Oncology":ti,ab,kw,de OR "Gynecologic Cancer":ti,ab,kw,de
	#2	"ovary tumor"/exp
	#3 Combine	#1 OR #2
	#4	"endometrium tumor"/exp
	#5	"Endometrial Neoplasms":ti,ab,kw,de OR "Endometrial Neoplasm":ti,ab,kw,de OR "Neoplasm, Endometrial":ti,ab,kw,de OR "Neoplasms, Endometrial":ti,ab,kw,de OR "Endometrial Carcinoma":ti,ab,kw,de OR "Carcinoma, Endometrial":ti,ab,kw,de OR "Carcinomas, Endometrial":ti,ab,kw,de OR "Endometrial Carcinomas":ti,ab,kw,de OR "Endometrial Cancer":ti,ab,kw,de OR "Cancer, Endometrial":ti,ab,kw,de OR "Cancers, Endometrial":ti,ab,kw,de OR "Endometrial Cancers":ti,ab,kw,de OR "Endometrium Cancer":ti,ab,kw,de OR "Cancer, Endometrium":ti,ab,kw,de OR "Cancers, Endometrium":ti,ab,kw,de OR "Cancer of the Endometrium":ti,ab,kw,de OR "Carcinoma of Endometrium":ti,ab,kw,de OR "Endometrium Carcinoma":ti,ab,kw,de OR "Endometrium Carcinomas":ti,ab,kw,de OR "Cancer of Endometrium":ti,ab,kw,de OR "Endometrium Cancers":ti,ab,kw,de OR "cervix neoplasia":ti,ab,kw,de
	#6	"uterine cervix tumor"/exp
	#7 Combine	#5 OR #6
	#8 Combine	"endometrioid carcinoma"/exp
	#9 Combine	"Carcinoma, Endometrioid":ti,ab,kw,de OR "Carcinomas, Endometrioid":ti,ab,kw,de OR "Endometrioid Carcinoma":ti,ab,kw,de OR "Endometrioid Carcinomas":ti,ab,kw,de OR "Adenocarcinoma, Endometrioid":ti,ab,kw,de OR "Adenocarcinomas, Endometrioid":ti,ab,kw,de OR "Endometrioid Adenocarcinomas":ti,ab,kw,de OR "Endometrioid Adenocarcinoma":ti,ab,kw,de OR "Endometrial Endometrioid Carcinoma":ti,ab,kw,de
	#10 Combine	#1 OR #2 OR #3 OR #4 OR #5 OR #6 OR #7 OR #8 OR #9

	#11 Combine	"tumor budding":ti,ab,kw,de OR "budding":ti,ab,kw,de OR "High-Grade Tumor Budding":ti,ab,kw,de OR "tumor-cell dissociation":ti,ab,kw,de
	#12 Combine	#10 AND #11

Data base	Search	Keywords
Cochrane Library	#1	"Gynecologic Oncology":ti,ab,kw OR "Gynecologic Cancer":ti,ab,kw
	#2	[mh "Ovarian Neoplasms"]
	#3 Combine	#1 OR #2
	#4	[mh "Endometrial Neoplasms"]
	#5	"Endometrial Neoplasms":ti,ab,kw OR "Endometrial Neoplasm":ti,ab,kw OR "Neoplasm, Endometrial":ti,ab,kw OR "Neoplasms, Endometrial":ti,ab,kw OR "Endometrial Carcinoma":ti,ab,kw OR "Carcinoma, Endometrial":ti,ab,kw OR "Carcinomas, Endometrial":ti,ab,kw OR "Endometrial Carcinomas":ti,ab,kw OR "Endometrial Cancer":ti,ab,kw OR "Cancer, Endometrial":ti,ab,kw OR "Cancers, Endometrial":ti,ab,kw OR "Endometrial Cancers":ti,ab,kw OR "Endometrium Cancer":ti,ab,kw OR "Cancer, Endometrium":ti,ab,kw OR "Cancers, Endometrium":ti,ab,kw OR "Cancer of the Endometrium":ti,ab,kw OR "Carcinoma of Endometrium":ti,ab,kw OR "Endometrium Carcinoma":ti,ab,kw OR "Endometrium Carcinomas":ti,ab,kw OR "Cancer of Endometrium":ti,ab,kw OR "Endometrium Cancers":ti,ab,kw
	#6	[mh "Uterine Cervical Neoplasms"]
	#7 Combine	#5 OR #6
	#8 Combine	[mh "Carcinoma, Endometrioid"]
	#9 Combine	"Carcinoma, Endometrioid":ti,ab,kw OR "Carcinomas, Endometrioid":ti,ab,kw OR "Endometrioid Carcinoma":ti,ab,kw OR "Endometrioid Carcinomas":ti,ab,kw OR "Adenocarcinoma, Endometrioid":ti,ab,kw OR "Adenocarcinomas, Endometrioid":ti,ab,kw OR "Endometrioid Adenocarcinomas":ti,ab,kw OR "Endometrioid

		Adenocarcinoma":ti,ab,kw OR "Endometrial Endometrioid Carcinoma":ti,ab,kw
	#10 Combine	#8 AND #9
	#11 Combine	"tumor budding":ti,ab,kw OR "budding":ti,ab,kw OR "High-Grade Tumor Budding":ti,ab,kw OR "tumor-cell dissociation":ti,ab,kw
	#12 Combine	#10 AND #11

Table S2. Main Characteristics of all gynecological cancer studies included studies

Site	Authors Year	Country	Sampling year	Age (Mean)	Pathological Stage	Follow up (Months)	Treatment	Pathologist Involvement (n)
Cervix	Huang,B et al 2016 [16]	China	2008-2014	45	IA2-IIA	37	Surgery	2
	Satabongkoch et al 2017 [71]	Thailand	2006-2012	45	IB2,IIB,IIA2	45	Surgery	4
	Jesinghaus et al 2018 [18]	Germany	1991-2016	47	-	89	-	3
	Park et al 2020 [19]	South Korea	2011-2015	49	IB2,IIB,IIA2,	60	Surgery	2
	Stanulović et al 2020 [24]	Serbia	2010-2018	59	-	-	-	-
	Zare et al 2020 [20]	Germany	2000-2018	45	IB2,IIB,IIA2	52	-	-
	Cao et al 2020 [25]	China	2009-2013	50	-	41	Surgery	2
Endometrium	Koyuncuoglu et al 2012 [21]	Turkey	1999-2009	61	IB2,IIB,IIA2,	49	Surgery	2
	Park et al 2019 [22]	South Korea	2008-2013	56	IB2,IIB,IIA2,	44	-	2
	Kluz et al 2020 [26]	Poland	2011-2014	63	IB1,IB2,2A	60	-	3
	Rau, T. T et al 2020 [25]	Switzerland	2004-2015	66	IB2,IIB,IIA2	-	-	2

Table S3. Characteristics of patients clinicopathological data included in the meta-analysis.

Author/Year	Koyuncuoglu 2012		Huang 2016		Satabongkoch 2017		Jesinghaus 2018		Park 2019		Kluz 2020		Stanulović 2020		Zare 2020	
Tumor budding (High-grade vs. Low-grade)	22	73	196	447	15	114	13	109	63	33	85	34	34	57	25	69
Age	-	-	-	-	5 (33.3%)	53 (46.5%)	9 (60%)	41 (37.6%)	-	-	-	-	-	-	16 (64%)	28 (40.5)
Stage (III and IV)	7 (31.8%)	9 (12.3%)	29 (14.7%)	48 (10.7%)	9 (60%)	31 (27.1%)	7 (53.8%)	19 (17.4%)	21 (33.3%)	22 (66%)	13 (15.2%)	4 (11.7%)	-	-	6 (64%)	8 (40.5%)
Depth of invasion (over than half)	-	-	-	-	9 (60%)	63 (55.26%)	-	-	38 (60.31)	8 (24.24)	-	-	-	-	-	-
N stage (N1, N2, N3)	-	-	58 (29.5%)	45 (1%)	7 (46.6%)	17 (14.9%)	7 (53.8%)	19 (17.4%)	33 (52.3%)	4 (12.1%)	-	-	22 (64.7%)	13 (22.8%)	14 (56%)	15 (21.7%)
M stage (M1)	-	-	10 (5.1%)	5 (1.1%)	-	-	-	-	-	-	-	-	-	-	1 (4%)	1 (1.4%)
Grade (G3)	10 (45.5%)	19 (26%)	77 (39.28%)	120 (26.8)	7 (46.6%)	9 (7.8%)	9 (60%)	67 (61.4%)	-	-	13 (15.3%)	1 (2.94%)	15 (44.1%)	5 (8.7%)	19 (76%)	44 (63.7%)
Lymphatic invasion (present)	5 (22.7%)	4 (5.5%)	76 (38.8%)	45 (1%)	12 (80%)	61 (53.5%)	13 (100%)	43 (39.4)	41 (65%)	6 (18.1%)	-	-	15 (44.1%)	18 (31.5%)	10 (40%)	29 (42%)
Perineural invasion (present)	-	-	6 (3%)	6 (1.3%)	-	-	7 (53.8%)	10 (0.91%)	-	-	-	-	8 (23.5%)	12 (21%)	7 (28%)	12 (17.3%)
